# Serum Immunoglobulins as Diagnostic Markers in Smokeless Tobacco Users for Prevention of Oral Potentially Malignant Disorders

**DOI:** 10.31557/APJCP.2020.21.7.2055

**Published:** 2020-07

**Authors:** Anjani Kumar Shukla, Tanya Khaitan, Prashant Gupta, Shantala R Naik

**Affiliations:** *Department of Oral Medicine and Radiology, Dental Institute, Rajendra Institute of Medical Sciences Ranchi, India. *

**Keywords:** Carcinogens, immunoglobulins, imperative, smokeless tobacco

## Abstract

**Background:**

Smokeless tobacco (SLT) remains a threat amongst a large population across the globe and particularly in India. Among the 28 known carcinogens in SLT, tobacco-specific nitrosamines are considered to be the most potent and it has been shown to cause immunomodulatory effects making the individual susceptible to various diseases. Immunoglobulins (Ig) form the defense against pathogens at the mucosal surfaces and SLT might interfere with its production and function. Therefore, the present study was undertaken to estimate the level of IgG and IgA in SLT patients and establish a correlation between them.

**Materials and Methods:**

A total of 60 subjects (34 khaini users and 26 gutkha users) were selected for the study. Complete demographic data and history was taken and clinical examination done to evaluate any oral mucosal changes. Venous blood samples were taken to analyze the serum immunoglobulin parameters.

**Results:**

Significant changes were observed in the serum IgA and IgG level in SLT users. Serum IgG level had a positive correlation whereas serum IgA had a negative correlation with the form of SLT and were statistically insignificant.

**Conclusion:**

The present study might serve as an early diagnostic tool and helpful in creating awareness on the hazards of using SLT among the Indian population as a despicable substitute to smoking tobacco. It also confers an imperative role into SLT mediated effects on immunoglobulins levels.

## Introduction

The use of smokeless tobacco (SLT) as drug substance has been used throughout the world although it has dangerous effect on human health (Rajasekhar et al., 2007). 20 per 100,000 populations are affected by oral cancer accounting for about 30% of all types of cancer in the country. The global burden of cancer continues to increase mostly because of increase in habits of tobacco, particularly smoke and smokeless forms (Coelho, 2012). 

World Health Organization South-East Asia region is domicile to 90% of global SLT users as over 250 million of such users live in this region (Sinha et al., 2012). During the first two decades of the 21st century, India is predicted to experience the fastest rate of rise in deaths attributable to tobacco worldwide. Given a population of over one billion people, this exponential increase in tobacco-related mortality from 1.4% of all deaths in India from 1990 to 13.3% in 2020 would result in tremendous social and economic burden for the country (Vikneshan et al., 2014).

 SLT are used in various forms in India such as pan (betel quid) with tobacco, zarda, pan masala, khaini, areca nut and slaked lime preparations, mawa, snuff, mishri and gudhaku. SLT products contain alkaloid nicotine and its principal metabolite cotinine. Besides the toxic chemicals like polycyclic aromatic hydrocarbons, nitrate, nicotine, acrolein, chemicals such as crotonaldehyde, formaldehyde, acetaldehyde, etc have also been reported (Biswas et al., 2015). 

Immunoglobulins are glycoproteins expressed as membrane bound receptors on the surface of B cells or soluble molecules secreted from plasma cells (Divya and Sathasivasubramanian, 2014). It has been demonstrated that mucosal immunity is depressed among tobacco smokers and chewers. Tobacco chewing affects a wide range of immunological functions in human and experimental animals including both humoral and cell mediated immune responses. According to Frial, nicotine activates dendritic cell and augments their capacity to stimulate T cell proliferation and cytokine secretion (Prajapati and Chawda, 2016). 

Very few studies on the effect of consumption of SLT on alteration in the levels of serum immunoglobulins have been reported in literature but no correlation has been established regarding the same. Considering the above background, the aim and objectives of the present study was to determine the effect of smokeless tobacco on serum major immunoglobulins (IgA and IgG) in SLT users and evaluate the correlation of form of SLT (khaini and gutkha) with serum immunoglobulins.

## Materials and Methods

The present study was initiated after approval of the institutional ethical committee. A total of 60 subjects (34 khaini users and 26 gutkha users) attending the outpatient department of Oral Medicine and Radiology, Dental Institute, RIMS, Ranchi were selected for the study. The participants enrolled in the study belonged to the age group of 20-70 years and were selected through simple random sampling technique. The refusal rate was found to be 6.2% (4 subjects refused to participate as they did not want to undergo any investigatory procedure) and not included in the study. All the subjects were being explained about the study and written informed consent obtained. Demographic data (including occupation and socio-economic status) was obtained for all individuals.

*Inclusion criteria*

Healthy individuals with history of consumption of SLT in any form were included in the study.

*Exclusion criteria*

Subjects with any systemic illness or immunocompromised conditions, those under alcohol consumption, smoking tobacco in any form and not willing to participate were excluded from the study.

The armamentarium consisted of diagnostic instruments, 5 ml syringe, vials containing ethylenediaminetetraacetic acid (EDTA), tourniquet, sterile cotton and surgical gloves. 5 ml of venous blood was collected from all subjects by using routine venipuncture method and stored in vials containing EDTA. Immunoglobulins A and G were analyzed using serum immunoturbidimetry by Horiba XL 80 at the hematology laboratory of the institution.

All the data obtained was noted in a proforma specially designed for the study. Comparison of the serum IgA and serum IgG in khaini and gutkha users was performed using t-test and spearman’s rank correlation coefficient with SPSS version 16.01 (statistical package for social sciences) software. Significance level was considered at 1% (p value <0.01) and 5% (p value <0.05).

## Results

A total of 60 subjects, 34 khaini users (32 males and 2 females) and 26 gutkha users (22 males and 4 females) with mean age of 36.9 years were selected for the study. Consumption of SLT was described in terms of duration (number of months/ years consumed) and frequency (number of times of consumption per day). When duration of the habit was being compared with khaini users, (8 khaini users) reported with <5 years, followed by 5-10 years (16 khaini users) and >10years (10 khaini users). The mean average of duration of khaini consumption was 11.29 years with frequency of 4 times /day. 

When duration of the habit was being compared with gutkha users, majority of the subjects (12 gutkha users) reported with <5 years, followed by 5-10 years (10 gutkha users) and >10years (4 gutkha users). The mean average of duration of gutkha consumption was 7.84 years with frequency of 7 times /day. 

The various oral mucosal changes observed were white lesion (26 khaini users) and (4 gutkha users), proliferative/ ulcerative growth (2 khaini users) and (8 gutkha users) mixed red and white lesion (2 gutkha users). Among white lesions, oral submucous fibrosis was seen predominantly in 16 gutkha users. Table 1 shows the distribution of oral mucosal changes seen in SLT users. No mucosal changes were observed in 2 gutkha users.

*Comparison of serum IgA in khaini users and gutkha users*

**Figure 1:**
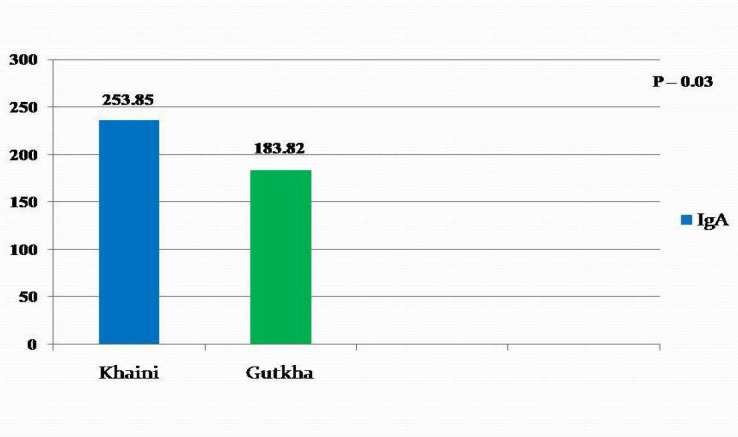
Comparison of Serum IgA in Khaini and Gutkha Users

Mean serum IgA was higher in khaini users (253.85 g/dl) when compared to gutkha users (183.82g/dl) which was statistically significant with a t value of 1.91 and p value of 0.03 (Figure 1).

*Comparison of serum IgG in khaini users and gutkha users*

**Figure 2:**
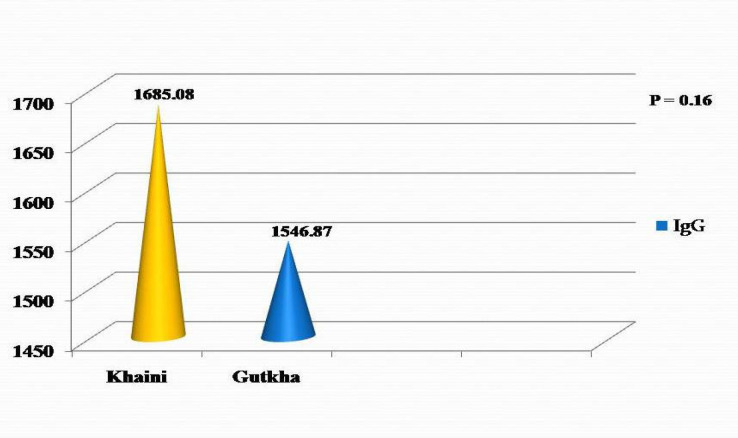
Comparison of Serum IgG in Khaini and Gutkha Users

Mean serum IgG was higher in khaini users (1685.08 g/dl) when compared to gutkha users (1546.87g/dl) which was statistically non-significant with a t value of 0.99 and p value of 0.16 (Figure 2).

*Correlation of form of serum IgA and IgG with oral mucosal change*s

Pearson’s correlation coefficient test was performed to correlate serum IgG and IgA and oral mucosal changes. Negative correlation was obtained but was statistically not significant with p value >0.01. [Table 2] This was indicative of the fact both the serum immunoglobulin markers were equally accountable for all the oral mucosal changes.

*Correlation of form of smokeless tobacco with oral mucosal changes, serum IgA and IgG parameters*

Pearson’s correlation coefficient test was performed to correlate the form of SLT and oral mucosal changes. Positive correlation was obtained with the form of SLT and oral mucosal changes but was statistically non-significant with p value of >0.01. Serum IgG level had a positive correlation with khaini and gutkha whereas serum IgA had a negative correlation and both were statistically non-significant (Table 3). This was suggestive of the fact that both khaini and gutkha had equally adverse systemic effects on these serum immunological markers.

**Table 1:** Distribution of Oral Mucosal Changes Seen in SLT Users According to Duration of Consumption

	Oral mucosal changes				
Variable	No mucosal changes	White lesion	Mixed red and white lesion	Proliferative/ Ulcerative growth	Total
Duration					
<5years	1	17	1	1	20
5-10years	1	15	1	9	26
>10years	0	14	0	0	14

**Table 2:** Correlation of Serum IgA and IgG Level with Oral Mucosal Changes

S. No	Serum Immunoglobulins	Pearson’s correlation (r)	*P*-value
1	IgA	-0.022	0.912 NS
2	IgG	-0.204	0.297 NS

NS, non-significant

**Table 3:** Correlation of Form of Smokeless Tobacco with IgA and IgG Level

S. No	Form of SLT	Pearson’s correlation (r)	*P*- value
1	Khaini	0.031	0.230 NS
2	Gutkha	-0.161	0.598 NS

NS, non-significant

## Discussion

The use of pan masala/gutkha is considered a benign and socially acceptable habit by most Indians. There is even an element of prestige associated with the habit. The addictive nature of tobacco compounds the problem, as quitting becomes difficult even for concerned users. This “socially accepted addiction” is, in fact, proving more dangerous than other addictions (Kumar et al., 2017). SLT consumption is more prevalent among lower socio-economic groups in India. It is believed to have stress relieving and healing properties for curing toothache, headache and stomach ache which dictates many adults to accede to its usage. Curiosity, peer pressure, offered by friends and acquaintances contribute to the initiation of its use (Gupta and Ray, 2003; Stepanov et al., 2006). Tobacco use is more common among males especially teenagers when compared with females. Male predominance with age group (30-40 yrs) was seen in the present study.

The tobacco-specific nitrosamines are considered to be the most potent among the 28 known carcinogens in SLT (National Cancer Institute, 1992), due to its strong carcinogenicity (Stepanov et al., 2006; Biswas et al., 2015). Chewing tobacco contains more than two dozen ingredients causing cancer; three to four times more nicotine than that delivered by a cigarette and stays for a longer time in the bloodstream. The rate of absorption may vary among different forms of smokeless tobacco depending on the pH level of the product, amount of nicotine and size of the tobacco cutting (Gupta et al., 2004).

Habit of chewing or holding of khaini and gutkha in the oral cavity allows absorption of nicotine and other carcinogens through oral mucosa. Orally absorbed nicotine increases the mucosal permeability of the tissues, thereby increasing its penetration, and the mucosa tries to defend the surface by increasing the defensive mechanism. As an early sign of damage to the oral mucosa, khaini and gutkha chewers often develop clinically visible whitish (leukoplakia) or reddish (erythroplakia) lesions and/or stiffening of the oral mucosa and oral submucous fibrosis which later transforms into malignancy (Kamath et al., 2013; Biswas et al., 2015; Ramya and Anuradha, 2015). All these oral mucosal changes were observed in the present study predominantly oral submucous fibrosis.

Serum immunoglobulins provide us the information about humoral immune status. Henceforth, a routine analysis of the same is very important. Low levels of immunoglobulins indicate certain immune deficiencies and high levels provide information about chronic inflammatory diseases, hematological disorders, infection and malignancies (Dispenzieri et al., 2001; Rakheerathnam et al., 2018). Five distinct classes of immunoglobulin molecules namely IgG, IgA, IgM, IgD and IgE are recognized in humans. IgG is the predominant immunoglobulin in serum (70-75%, approximately 1000 mg/dl). Secretory IgA is the next most predominant immunoglobulin, accounting for approximately 15-20% (approximately 200 mg/dl). Previous studies are suggestive of the fact that nicotine is the major immunosuppressive in cigarette and/or smokeless tobacco. Nicotine causes the secretion of catecholamines that have suppressive effects on immune system by inducing adrenocorticotropic hormone secretion (Aral et al., 2006). The possible mechanism behind increased levels of immunoglobulins might be that tobacco impairs the immunologic functions of the body carried out by immunoglobulins (Prajapati and Chawda, 2016).

Increase in IgG level was seen in SLT users when compared to the normal range in the present study. It was higher in khaini users when compared to gutkha users and was statistically non-significant. IgG is considered as the major antibody in secondary antibody response. Continuous exposure of oral mucosa to the SLT components could mediate a stimulatory effect on immunoglobulin production, and thus the observed elevated level in the present study (Rakheerathnam et al., 2018). This could also be due to chronic inflammation in SLT users. Similar increase in IgG level was seen in study conducted by (Pinakapani et al., 2009; Patidar et al., 2011). In contrary, IgG level was decreased in study conducted by Rajendran et al., (1986).

IgA is mainly found in the mucosal secretions. It also circulates in blood and its main function in circulation is to clear immune complexes by phagocytosis. It has been reported that the production of IgA is regulated by transforming growth factor (TGF)-β, a multifunctional cytokine. The TGF-β is reported to play potential roles, such as growth, differentiation, and modulation of immune responses (Rakheerathnam et al., 2018). The serum IgA levels in the present study was within the normal range. IgA level was higher in khaini users when compared to gutkha users and was statistically significant. In contrary, IgA level was increased in study conducted by Kalpana et al., (2011) and Prajapati and Chawda (2016). This is the first study in our knowledge suggesting that IgA plays no substantial role on SLT users although it is slightly higher in khaini users.

In the tobacco habituals, the toxins are liberated by tobacco from bidi/cigarette smoke and arecoline from gutkha. The role of IgG in the body is to neutralize such toxins therefore causing an increase in the level of IgG as a defensive mechanism in such patients (Divya et al., 2014). To substantiate this, Pearson’s correlation was performed to correlate the form of SLT and serum IgG and IgA. Serum IgG level had a positive correlation with khaini and gutkha whereas serum IgA had a negative correlation and both were statistically non significant indicative of the fact that both khaini and gutkha had equally adverse systemic effects on these serum immunological markers. Previously done researches suggest no such correlation between the form of SLT and serum immunological markers, thus highlighting the importance of our study.

In conclusion, immune profile is beneficial in early detection, management and prognosis of both the groups of patients in mass screening program, as khaini and gutkha chewing habits are more prevalent at present. The present study might serve as an early diagnostic tool in any systemic diseases and be helpful in spreading awareness on the deleterious effect of using SLT among the Indian population as a despicable alternative to tobacco smoke. Awareness campaigns carried out among youths regarding the deleterious effect of tobacco may lower the adverse effects. Research into immunological aspects is becoming an excellent model for studying genetic-environmental-immunologic-nutritional interactions in disease pathogenesis.
